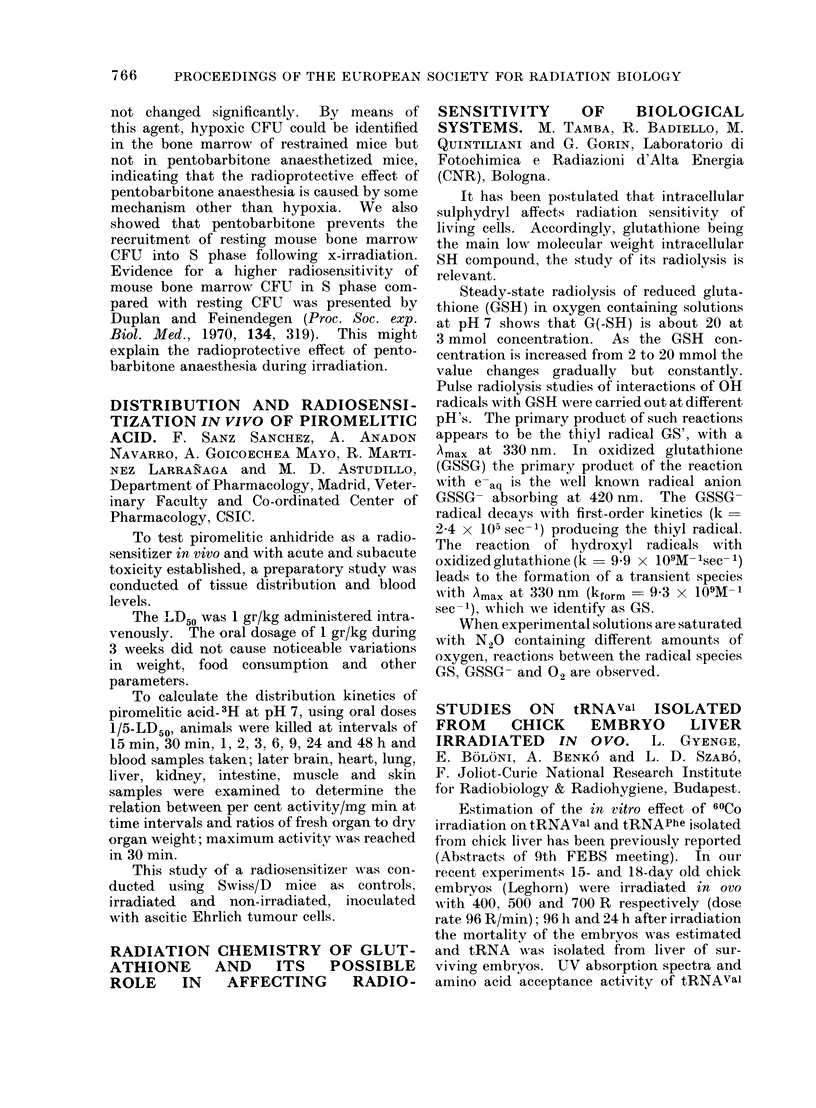# Proceedings: Radiation chemistry of glutathione and its possible role in affecting radio-sensitivity of biological systems.

**DOI:** 10.1038/bjc.1975.339

**Published:** 1975-12

**Authors:** M. Tamba, R. Badiello, M. Quintiliani, G. Gorin


**RADIATION CHEMISTRY OF GLUTATHIONE AND ITS POSSIBLE ROLE IN AFFECTING RADIOSENSITIVITY OF BIOLOGICAL SYSTEMS.** M. TAMBA, R. BADIELLO, M. QUINTILIANI and G. GORIN, Laboratorio di Fotochimica e Radiazioni d'Alta Energia (CNR), Bologna.

It has been postulated that intracellular sulphydryl affects radiation sensitivity of living cells. Accordingly, glutathione being the main low molecular weight intracellular SH compound, the study of its radiolysis is relevant.

Steady-state radiolysis of reduced glutathione (GSH) in oxygen containing solutions at pH 7 shows that G(-SH) is about 20 at 3 mmol concentration. As the GSH concentration is increased from 2 to 20 mmol the value changes gradually but constantly. Pulse radiolysis studies of interactions of OH radicals with GSH were carried out at different pH's. The primary product of such reactions appears to be the thiyl radical GS', with a $\lambda_{max}$ at 330 nm. In oxidized glutathione (GSSG) the primary product of the reaction with $e^-_{aq}$ is the well known radical anion $GSSG^-$ absorbing at 420 nm. The $GSSG^-$ radical decays with first-order kinetics ($k = 2{\cdot}4 \times 10^5\ sec^{-1}$) producing the thiyl radical. The reaction of hydroxyl radicals with oxidized glutathione ($k = 9{\cdot}9 \times 10^9 M^{-1} sec^{-1}$) leads to the formation of a transient species with $\lambda_{max}$ at 330 nm ($k_{form} = 9{\cdot}3 \times 10^9 M^{-1} sec^{-1}$), which we identify as GS.

When experimental solutions are saturated with $N_2O$ containing different amounts of oxygen, reactions between the radical species GS, $GSSG^-$ and $O_2$ are observed.